# The association of essential dietary trace elements and their mixture with cognition: a prospective study

**DOI:** 10.3389/fnut.2025.1461852

**Published:** 2025-07-04

**Authors:** Huihui Li, Zhongmin Yin, Fusheng Cui, Weijing Wang, Dongfeng Zhang

**Affiliations:** ^1^Department of Epidemiology and Health Statistics, Public Health College, Qingdao University, Qingdao, China; ^2^Department of Outpatient and Emergency, The Affiliated Hospital of Qingdao University, Qingdao, China

**Keywords:** BKMR, cognition, essential trace element, old adults, UK Biobank

## Abstract

**Background:**

The association of dietary essential trace elements (ETEs) and their mixture with cognition remains unclear.

**Methods:**

Prospective cohort data on the association between dietary ETEs [e.g., iron (Fe), copper (Cu), zinc (Zn), manganese (Mn), selenium (Se), and iodine (I)] and general cognition were obtained from the UK Biobank (UKB) database. Linear regression and restricted cubic splines (RCS) were used to examine the association between individual dietary ETEs and general cognition, including the identification of the inflection points. The Bayesian kernel machine regression (BKMR) model was applied to analyze the association between a mixture of six ETEs and general cognition, as well as potential interactions among ETEs. Stratified analysis and sensitivity analysis were also conducted.

**Results:**

Significant non-linear association between individual dietary ETEs and general cognition was observed, with the inflection points for the various elements being as follows: Fe: 15 mg/day, Zn: 10 mg/day, Cu: 1.5 mg/day, I: 250 μg/day, Mn: 5 mg/day, and Se: 45 μg/day. The BKMR analysis showed an inverted “U”-shaped association between dietary ETE mixture and general cognition, with Fe and Zn playing major roles. Dietary Mn was the major contributor in males, while Zn was predominant in females. In the hypertensive population, dietary Zn and Mn play major roles.

**Conclusion:**

There are non-linear associations between dietary Fe, Zn, Cu, I, Mn, Se—as well as their mixture—and general cognition. Among these, Fe and Zn play major roles within this mixture. In addition, there are sex differences in the main contributing ETE, with Mn in males and Zn in females.

## Introduction

As the aging population continues to grow, age-related cognitive decline has become an important public health challenge. When cognitive decline progresses more rapidly than expected for an individual’s age and education, mild cognitive impairment (MCI) can occur (1). MCI can have a significant effect on daily life and significantly increase the risk of dementia (2). According to a report by a 2021 report by the World Health Organization (WHO), the number of people living with dementia is predicted to reach 150 million by 2025, posing a huge social and economic burden (3). However, current pharmacological treatments for dementia can only slow disease progression rather than cure it and are not effective for all patients. Therefore, there is an urgent need to prioritize preventive strategies to delay cognitive decline.

Diet has been shown to have a major impact on cognitive function (4–7). Dietary essential trace elements (ETEs), absorbed through the gastrointestinal tract, can influence cognition by participating in enzyme synthesis, antioxidant systems, and immune system functions, and also by exerting neurotoxic effects (8–13). Previous epidemiology studies have explored the associations between single dietary ETE and cognition. As an example, dietary iron (Fe) intake was found to have a negative correlation with cognition in Li’s study, yet Liu J et al. observed a U-shaped relationship between Fe and dementia risk (8, 14). Dietary zinc (Zn) was found to have an L-shaped association with cognition among the older Chinese population, while such an association was not observed among Americans (15, 16). Similarly, Lorenzo-Mora Am et al. observed a positive correlation between high intake of manganese (Mn) and cognition, while a negative correlation was observed in another study (17, 18). Ke Jiang et.al reported that high selenium (Se) intake was related to better cognition, while the opposite result was observed in an NHANES study (8, 19, 20). Previous studies on single dietary ETEs and cognition were mainly conducted based on a cross-sectional study design with a small sample size, and hence, the ability of causal inference was weak. Moreover, existing studies have insufficiently explored the non-linear relationships between individual ETEs and cognition, with the inflection points of these associations remaining unclear.

Studies have shown that dietary ETEs may interact with each other during absorption. A higher dietary intake of Zn can inhibit the absorption of copper (Cu), and vice versa (21, 22). Elevated Zn levels can also inhibit the absorption of Fe (23). Considering the presence of multiple ETEs in the diet and their competitive interactions during absorption (24–26), it is crucial to analyze the association between dietary ETE mixture and cognition. The Bayesian Kernel Machine Regression (BKMR) is a powerful statistical machine learning algorithm that can effectively handle interactions between exposure variables and further explore the overall effects of the mixture (27). Previous studies have applied the BKMR method to analyze the association of ETEs in whole blood and urine with cognition among the Chinese population and found it to be a linear positive association (28, 29). However, there is a lack of studies using BKMR to investigate the association between the dietary ETE mixture and cognition. Furthermore, existing studies have primarily focused on older Chinese populations, with other ethnic groups remaining understudied.

In this study, based on the prospective cohort data from the UK Biobank (UKB), we explored the association of six individual dietary ETEs [Fe, Cu, Zn, Mn, Se, and iodine (I)] with cognition and further identified the inflection points in the associations to find the optimal intake range. Next, we analyzed the association between the mixture of six dietary ETEs and cognition, as well as the relative importance of each ETE on cognition and the potential interactions among them, using the BKMR model.

## Methods

### Study population

The UKB is a population-based prospective database that collects biological samples, lifestyle and health information, and other data from over 500,000 participants across the UK to support research aimed at improving public health. All participants provided informed consent, and the project was approved by the Northwest Multi-Centre Research Ethics Committee (reference 06/MRE08/65).

This study excluded participants with missing cognitive test data (*N* = 187,743) and missing covariate data (*N* = 2,964) from a total of 121,948 participants with at least one set of 24-h dietary recall data. We also excluded participants with dementia at baseline (*N* = 2). Finally, a total of 21,356 participants were included in this study. Supplementary Figure S1 shows the detailed screening process.

### Dietary essential trace elements intake assessment

The UKB collected five 24-h dietary recall data through online questionnaires, from April 2009 to June 2012, and calculated the nutrient intake based on the questionnaire data (30). This study analyzed six dietary ETEs, including Fe, Zn, Cu, I, Mn, and Se. The mean intake of these six ETEs was calculated for participants with at least one set of 24-h dietary recall data. Additionally, we performed *log*(X + 1) transformation and standardization of the intake of ETEs to control skewed distributions and differences in measurement units.

### Cognitive function tests

Cognitive test data were collected through touchscreen questionnaires at four assessment centers starting in 2014. This study primarily used five cognitive test data: reaction time (Field ID: 20023, log-transformed), numeric memory (Field ID: 4285), fluid intelligence/reasoning (Field ID: 20128), prospective memory (Field ID: 20018), and pair matching (Field ID: 399, log(x + 1) transformed). Detailed descriptions of the cognitive tests are provided in Supplementary Table S1. After appropriate transformation for skewed distributions, these five cognitive test data were entered into a principal component analysis (PCA), with the first unrotated principal component score being used as a measure of general cognition, where higher scores indicated better cognition (31). The study by Fawns-Ritchie et al. showed a high correlation between general cognition measured using only five baseline tests from the UKB and general cognition measured using a battery of standard cognitive assessments (32, 33). Therefore, the general cognition derived from the five cognitive tests in the UKB reliably and effectively reflected the cognitive performance of the participants.

### Covariates

Covariates included sociodemographic variables: age, sex (male, female), ethnicity (white, others), education level (college, not-college), employment status (yes, no), Townsend deprivation index (TDI), and body mass index (BMI, kg/m^2^); lifestyle factors: smoking status (never, previous, current), alcohol status (never, previous, current), physical activity level (low, moderate, high), and total dietary energy intake (kJ/day); and health conditions: hypertension (yes, no), diabetes (yes, no), and cardiovascular disease (yes, no). The BMI was calculated as weight (kg) / height (m)^2^. Physical activity level was categorized based on the metabolic equivalents outlined in the International Physical Activity Questionnaire (IPAQ) guidelines. Hypertension was defined using a combination of self-reported medical history, medication use, and diagnostic codes from electronic health records (ICD-9 and ICD-10). Other health conditions were defined based on self-reported information and electronic health record data. Detailed descriptions of these conditions are provided in Supplementary Table S2.

### Statistical analysis

This study divided participants into low cognition (<=0) and high cognition (>0) groups based on the average value of general cognition. For continuous variables, *t*-tests and Spearman’s rank correlation coefficient were used to compare the differences between the two groups. For categorical variables, Spearman’s rank correlation coefficient, chi-squared test, and Fisher’s exact test were used to estimate the bivariate correlation of dietary ETEs. After grouping the dietary ETEs into quartiles, with the lowest quartile as the reference group, we conducted a multiple linear regression to assess the association between single dietary ETE and general cognition. Additionally, restricted cubic splines (RCS) were used to explore the non-linear associations between ETEs and cognition, in an attempt to determine the inflection points.

We also constructed a BKMR model with 10,000 iterations to assess the association between a mixture of dietary ETEs and general cognition. Additionally, we calculated the posterior inclusion probabilities (PIPs) to assess the relative contributions of dietary ETEs to general cognition. The results of BKMR analysis included: (1) the overall effects of the mixture of dietary ETEs on general cognition compared to the median; (2) single-exposure effect of a specific ETE on general cognition when the remaining ETEs were controlled at the 25th, 50th, and 75th percentiles, respectively; (3) the univariate exposure–response relationship between a specific ETE and general cognition when the remaining ETEs were controlled; and (4) bivariate exposure–response functions for each ETE when the second ETE was fixed at a different level (10th, 50th, and 90th percentiles) and the remaining ETEs were fixed at their medians. Due to the sex differences in the risk of dementia, we conducted stratified analyses based on sex (34). As hypertension is a prominent risk factor for cognitive decline (35), we repeated the analyses in the hypertension subgroup.

Sensitivity analyses were also performed. As over 97% of the participants were White, we conducted the sensitivity analysis after excluding non-White participants. Next, we excluded participants with a history of hypertension, diabetes, and cardiovascular disease, attempting to focus on a relatively healthy population. Participants who developed dementia during the follow-up period (*N* = 15) were then excluded. Finally, general cognition was redefined based on the 11 cognitive tests in the UKB.

The statistical analysis was conducted using R.2.3. Statistical significance was set at a two-tailed *p*-value of < 0.05.

## Results

A total of 21,356 participants were included in this study. The basic characteristics of the participants are shown in Table 1. In comparison to individuals with higher general cognition, those with lower general cognition were more likely to be male, older, have a smoking and drinking history, be unemployed, and have lower education levels. Additionally, they were more likely to have a history of hypertension, diabetes, and cardiovascular disease.

**Table 1:** Characteristics of participants grouped by general cognition (*N* = 21,356).

Characteristics	Overall	Low cognition	High cognition	*p*
	(*N* = 21,356)	(*N* = 10,391)	(*N* = 10,685)	
Age (median [IQR])	55.0 [49.0, 61.0]	57.0 [51.0, 62.0]	53.0 [47.0, 59.0]	<0.001
Sex (%)				<0.001
Male	10,833 (50.7)	5,578 (53.7)	5,255 (47.9)	
Female	10,523 (49.3)	4,813 (46.3)	5,710 (52.1)	
Smoke (%)				<0.001
Never	13,087 (61.3)	6,205 (59.7)	6,882 (62.8)	
Previous	7,062 (33.1)	3,614 (34.8)	3,448 (31.4)	
Current	1,207 (5.7)	572 (5.5)	635 (5.8)	
Alcohol (%)				0.128
Never	453 (2.1)	238 (2.3)	215 (2.0)	
Previous	439 (2.1)	227 (2.2)	212 (1.9)	
Current	20,464 (95.8)	9,926 (95.5)	10,538 (96.1)	
BMI kg/m^2^ (mean (SD))	26.4 (4.2)	26.4 (4.2)	26.3 (4.2)	0.068
TDI (median [IQR])	−2.6 [−3.9, −0.5]	−2.6 [−3.9, −0.4]	−2.7 [−3.9, −0.5]	0.01
Physical activity group (%)				<0.001
Low	3,907 (18.3)	1730 (16.6)	2,177 (19.9)	
Moderate	9,179 (43.0)	4,407 (42.4)	4,772 (43.5)	
High	8,270 (38.7)	4,254 (40.9)	4,016 (36.6)	
Education (%)				<0.001
College	12,371 (57.9)	5,483 (52.8)	6,888 (62.8)	
Not-college	8,985 (42.1)	4,908 (47.2)	4,077 (37.2)	
Employment (%)				<0.001
No	6,240 (29.2)	3,656 (35.2)	2,584 (23.6)	
Yes	15,116 (70.8)	6,735 (64.8)	8,381 (76.4)	
Total energy kJ/day (mean (SD))	8800.55 (2394.71)	8728.61 (2451.62)	8868.74 (2337.57)	<0.001
Race (%)				<0.001
White	20,797 (97.4)	10,026 (96.5)	10,771 (98.2)	
Not white	559 (2.6)	365 (3.5)	194 (1.8)	
History of hypertension (%)				<0.001
No	16,554 (77.5)	7,817 (75.2)	8,737 (79.7)	
Yes	4,802 (22.5)	2,574 (24.8)	2,228 (20.3)	
History of diabetes (%)				<0.001
No	20,749 (97.2)	10,047 (96.7)	10,702 (97.6)	
Yes	607 (2.8)	344 (3.3)	263 (2.4)	
History of cardiovascular disease (%)				<0.001
No	17,042 (79.8)	8,065 (77.6)	8,977 (81.9)	
Yes	4,314 (20.2)	2,326 (22.4)	1988 (18.1)	

IQR, interquartile range; SD, standard deviation.

Table 2 shows the results of the linear regression of dietary ETEs with general cognition. Compared with the lowest quartile (Quartile 1), Quartiles 2, 3, and 4 of Fe are associated with higher general cognition. Additionally, Quartiles 2 and 3 of Zn, Cu, and Se are also associated with higher general cognition. In the linear regression results of dietary ETEs with specific cognitive domains, significant associations were rarely seen (Supplementary Table S3).

**Table 2:** Associations between single dietary essential trace element (ETE) intake and general cognition.

Essential trace elements	Quartile 2#	Quartile 3#	Quartile 4#
	β (95%CI)	β (95%CI)	β (95%CI)
Fe	**0.121 (0.075, 0.167)**	**0.106 (0.057, 0.155)**	**0.092 (0.033, 0.152)**
Zn	**0.077 (0.032, 0.123)**	**0.059 (0.011, 0.107)**	−0.028 (−0.084, 0.027)
Cu	**0.057 (0.011, 0.103)**	**0.110 (0.060, 0.159)**	0.021 (−0.036, 0.077)
I	0.039 (−0.006, 0.084)	0.019 (−0.027, 0.066)	0.020 (−0.029, 0.068)
Mn	0.021 (−0.024, 0.066)	0.008 (−0.039, 0.055)	−0.030 (−0.081, 0.021)
Se	**0.124 (0.079, 0.168)**	**0.087 (0.041, 0.133)**	0.043 (−0.005, 0.090)

#, the quartile 1 of dietary essential trace elements serves as a reference.

95%CI: 95% confidence interval.

Adjusted for age, sex, ethnicity, smoking, alcohol, BMI, TDI, physical activity, total energy, diabetes, hypertension, cardiovascular disease, education, and employment.

Statistically significant results were highlighted in bold.

**Figure 1:**
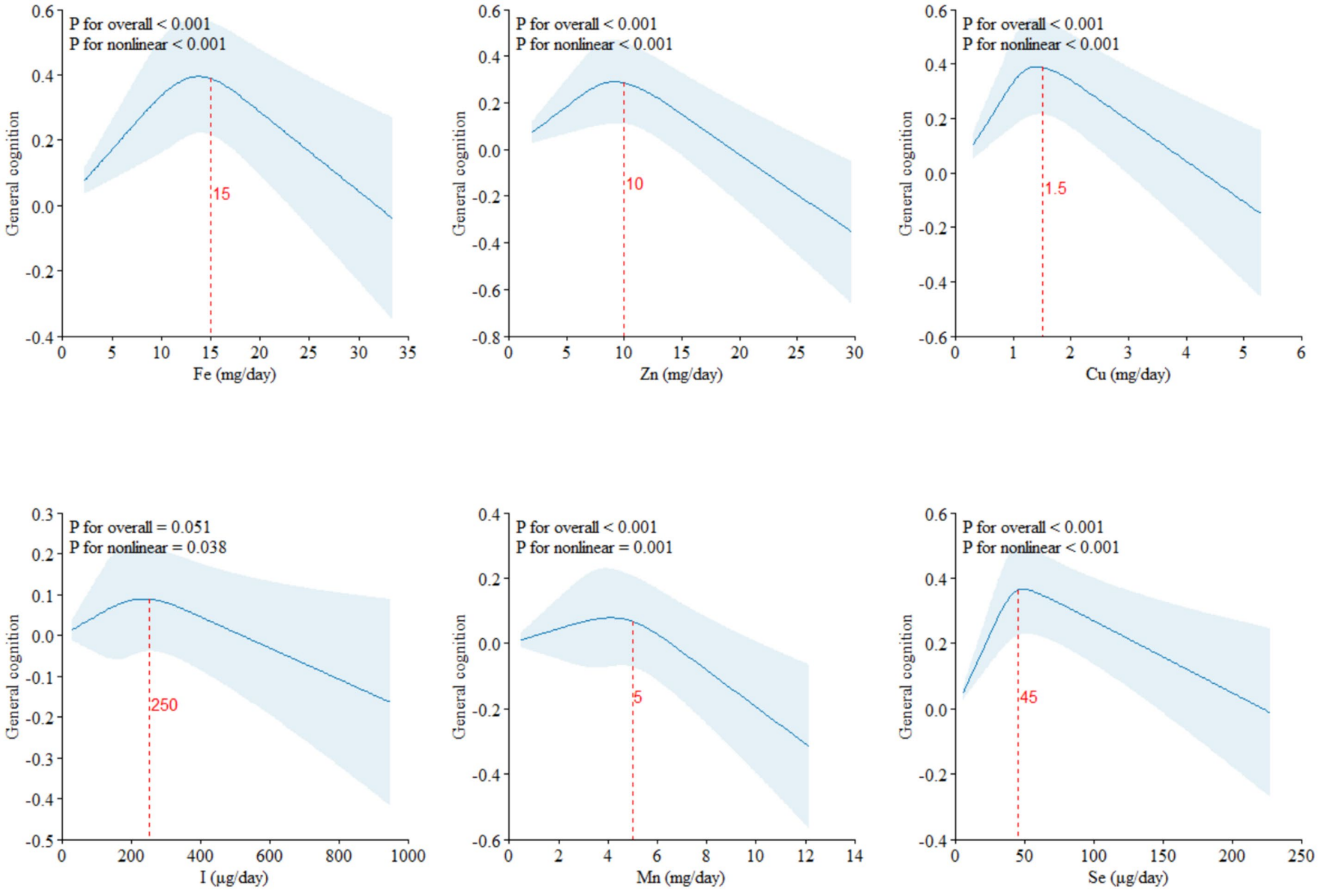
Restricted cubic splines (RCS) model of the dose–response associations between dietary ETE intake and cognitive function (*N* = 21,356). Dietary ETE intake without transformation and standardization; Fe, iron; Zn, zinc; Cu, copper; I, iodine; Mn, manganese; Se, selenium. Adjusted for age, sex, ethnicity, smoking, alcohol, BMI, TDI, physical activity, total energy, diabetes, hypertension, cardiovascular disease, education, and employment.

The RCS results of dietary ETEs with general cognition are shown in Figure 1. Fe (*P*_for non-linear_ < 0.001), Zn (*P*_for non-linear_ < 0.001), Cu (*P*_for non-linear_ < 0.001), I (*P*_for non-linear_ = 0.038), Mn (*P*_for non-linear_ = 0.001), and Se (*P*_for non-linear_ < 0.001) exhibited significant non-linear associations with general cognition. With increasing dietary intake, general cognition showed a trend of initially increasing and then decreasing. The inflection points for Fe, Zn, Cu, I, Mn, and Se were approximately at 15 mg/day, 10 mg/day, 1.5 mg/day, 250 μg/day, 5 mg/day, and 45 μg/day, respectively.

**Figure 2:**
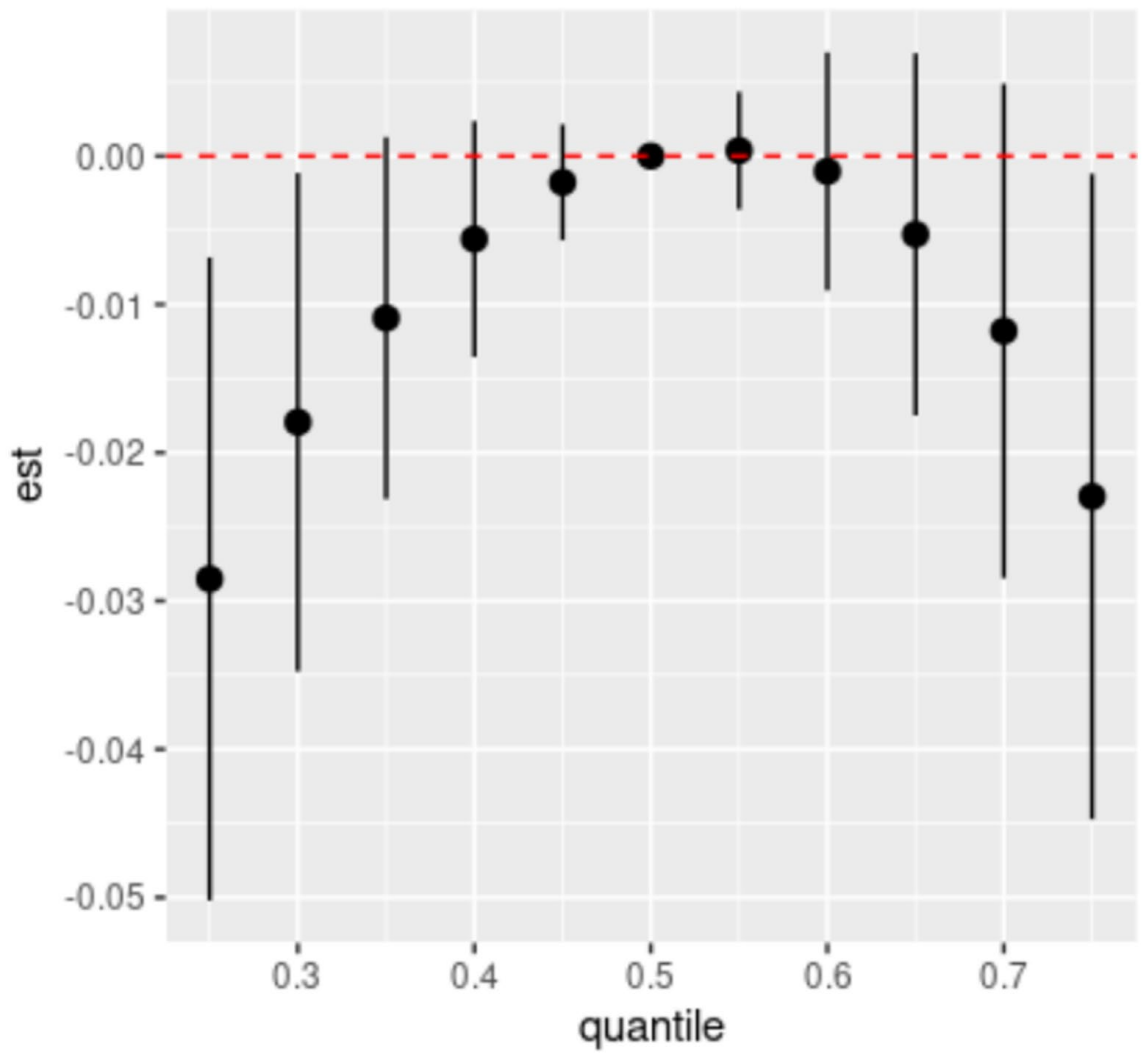
Overall effects of dietary ETE mixture on general cognition. The estimated changes and 95% credible intervals are represented by black dots with corresponding error bars. These values indicate the change in general cognition when all dietary ETEs are at different percentiles compared to when they are fixed at their 50th percentile. Dietary mineral intakes are *log*(x + 1)-transformed and standardized. Adjusted for age, sex, ethnicity, smoking, alcohol, BMI, TDI, physical activity, total energy, diabetes, hypertension, cardiovascular disease, education, and employment. Quantile, quantile of dietary ETE mixture; est., estimated cognitive function changes.

**Figure 3:**
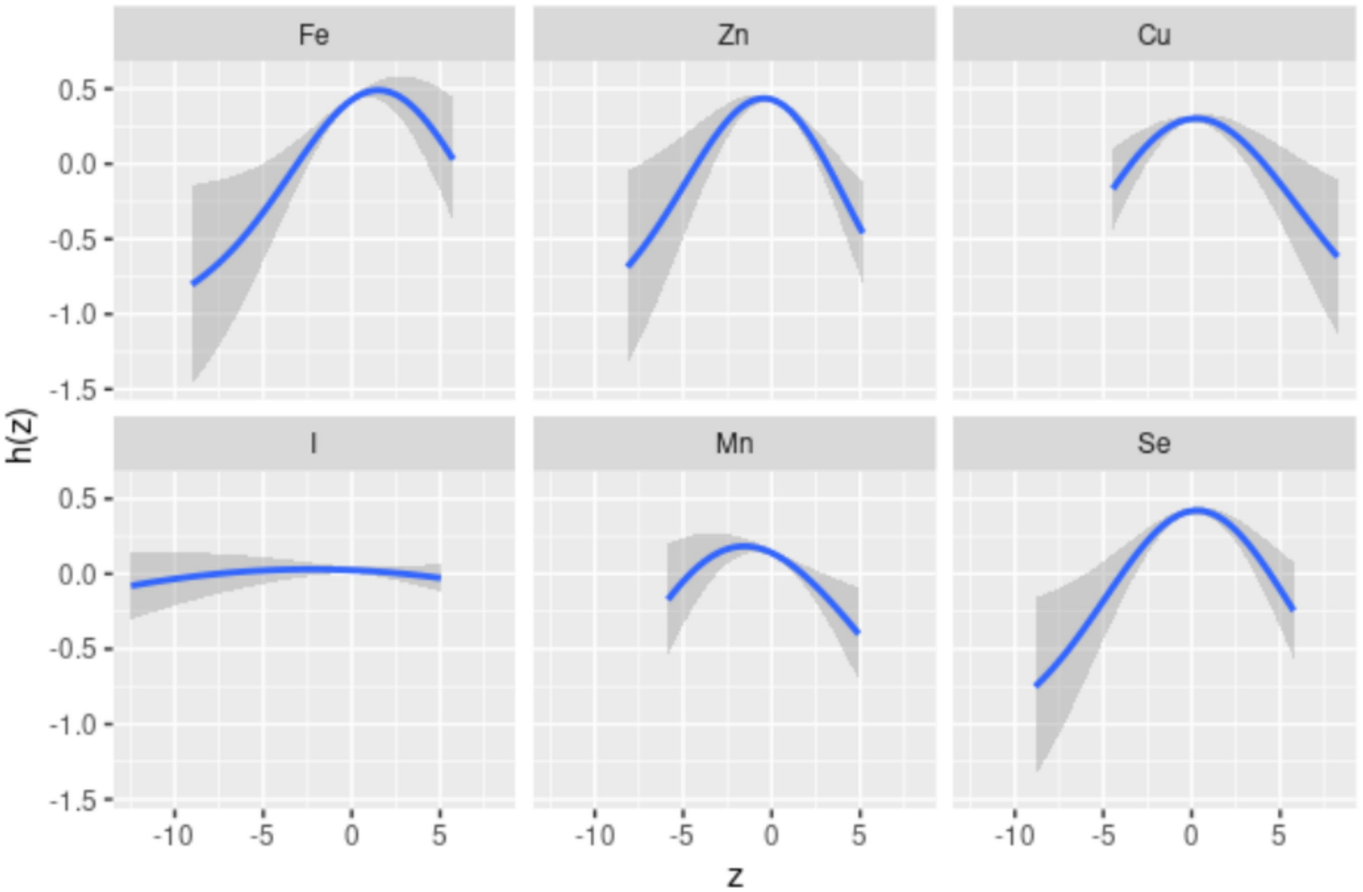
Univariate exposure–response function. Univariate exposure–response function and 95% credible interval (shaded areas) for each dietary ETE, when other ETEs are fixed at their 50th percentile. Dietary ETE intakes are log(x + 1)-transformed and standardized. Adjusted for age, sex, ethnicity, smoking, alcohol, BMI, TDI, physical activity, total energy, diabetes, hypertension, cardiovascular disease, education, and employment. z, the quantile of single dietary ETE; h(z), an exposure–response function that can accommodate non-linear exposure–outcome relationships.

Spearman’s correlation analysis (Supplementary Figure S2) shows low to moderate correlations (*r* values ranging from 0.51 to 0.87) among the six dietary ETEs. Figure 2 indicates that compared to the 50th percentile, there is a significant decline in general cognition when the intake of all dietary ETEs was below the 30th percentile or above the 75th percentile. Supplementary Table S4 shows that Fe and Zn had the highest PIP values at 1.000, followed by Mn (0.998), Cu (0.980), Se (0.954), and I (0.056). The single-exposure effect suggested that when the other dietary ETE intake was fixed at the 25th, 50th, or 75th percentile, the change in single dietary ETE intake increased from the 25th to the 75th percentile (Supplementary Figure S3). The exposure–response function results (Figure 3) showed inverted “U”-shaped associations between Fe, Zn, Cu, Mn, and Se and general cognition when the other dietary ETE intake was fixed at the median, while no significant correlation was found between I and general cognition. The results of interactions (Supplementary Figure S4) suggested a potential interaction between Fe and Zn as well as Mn and Zn, indicating a stronger impact of Zn on general cognition when Fe and Mn intake increased from the 10th to the 50th percentile.

**Figure 4:**
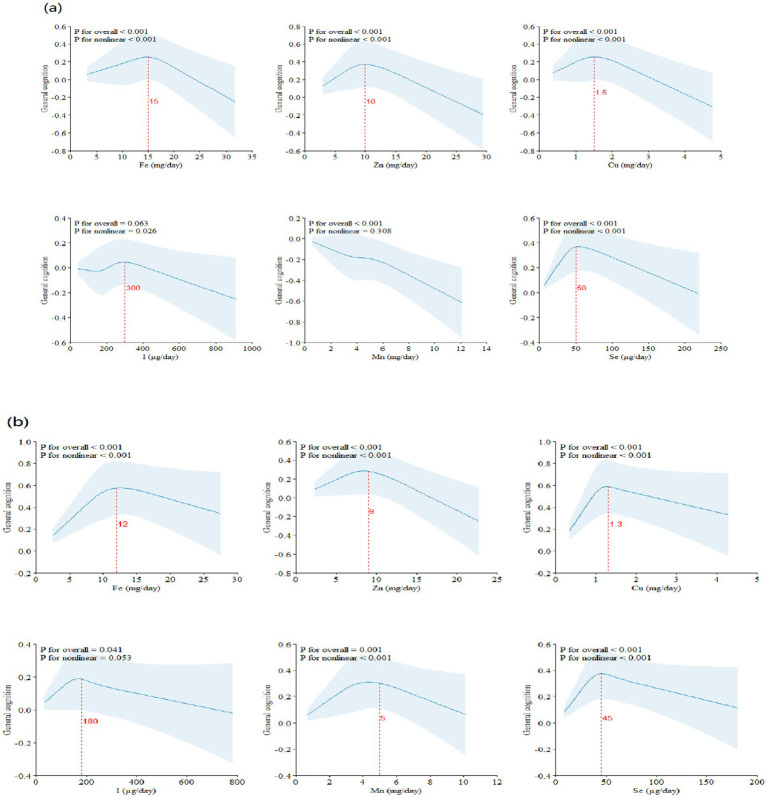
RCS model of the dose–response association between dietary ETE intake and cognitive function stratified by sex (**a**, males; **b**, females) (*N* = 21,356). Dietary ETE intake without transformation and standardization; Fe, iron; Zn, zinc; Cu, copper; I, iodine; Mn, manganese; Se, selenium. Adjusted for age, sex, ethnicity, smoking, alcohol, BMI, TDI, physical activity, total energy, diabetes, hypertension, cardiovascular disease, education, and employment.

**Figure 5:**
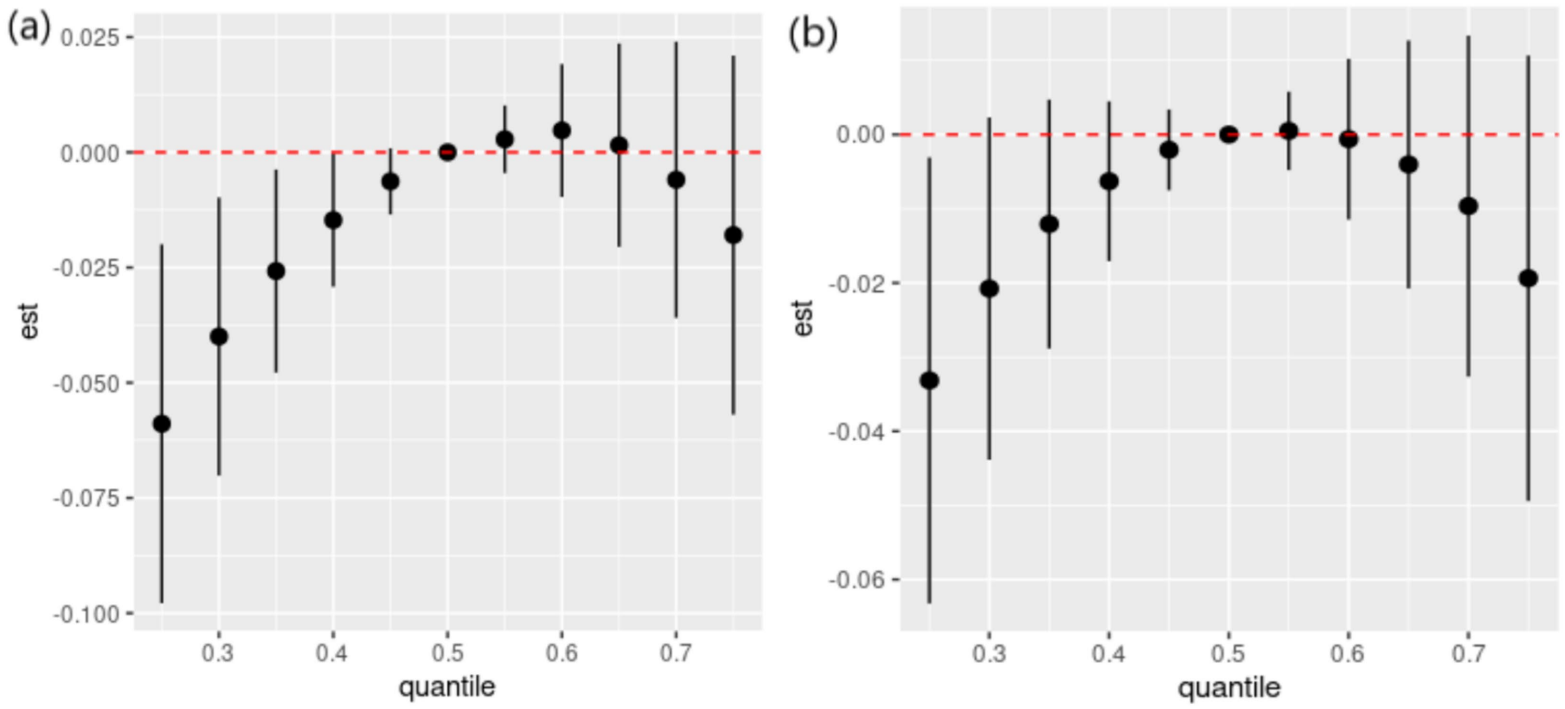
Overall the effects of dietary ETEs on general cognition stratified by sex (**a**, males; **b**, females). Adjusted for age, sex, ethnicity, smoking, alcohol, BMI, TDI, physical activity, total energy, diabetes, hypertension, cardiovascular disease, education, and employment. Quantile, quantile of dietary ETE mixture; est., estimated cognitive function changes.

**Figure 6:**
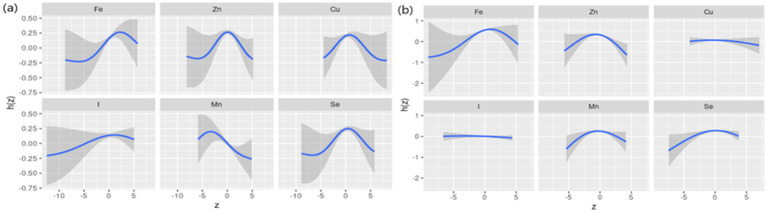
Univariate exposure–response function stratified by sex (**a**, males; **b**, females). Univariate exposure–response function and 95% credible interval (shaded areas) for each dietary ETE, when other ETEs were fixed at their 50th percentile. z, the quantile of single dietary ETE; h(z), an exposure–response function that can accommodate non-linear exposure–outcome relationships. Dietary ETE intakes are log(x + 1)-transformed and standardized. Adjusted for age, sex, ethnicity, smoking, alcohol, BMI, TDI, physical activity, total energy, diabetes, hypertension, cardiovascular disease, education, and employment.

The results of the interaction effects between dietary ETEs and sex are shown in Supplementary Table S5. We did not observe significant interactions between dietary ETEs and sex, except for Mn. However, given the importance of sex-related dementia risk, we conducted a stratified analysis based on sex. The results of sex-stratified analyses are shown in Figures 4–6. Sex-related variations were observed in the associations of Fe (males: 12 mg/day, females: 15 mg/day), I (males: 180 μg/day, females: 300 μg/day), and Mn (males: 5 mg/day, females: not detected) with general cognition (Figure 4). The trends in the overall effects of dietary ETEs on general cognition were consistent with the primary result in both males and females (Figure 5). The highest PIP was Mn (1.000) in males, while it was Zn (1.000) in females. In Supplementary Figure S5, an increase in dietary Mn intake in males was associated with a decline in general cognition when the other dietary ETEs were fixed at the 25th, 50th, or 75th percentile. For females, an increase in dietary Zn intake was associated with a decline in general cognition when the other dietary ETEs were fixed at the 25th or 50th percentile. Non-linear associations were observed between Fe, Zn, Cu, Mn, Se, and general cognition in males, and between Fe, Zn, Mn, Se, and general cognition in females (Figure 6).

**Figure 7:**
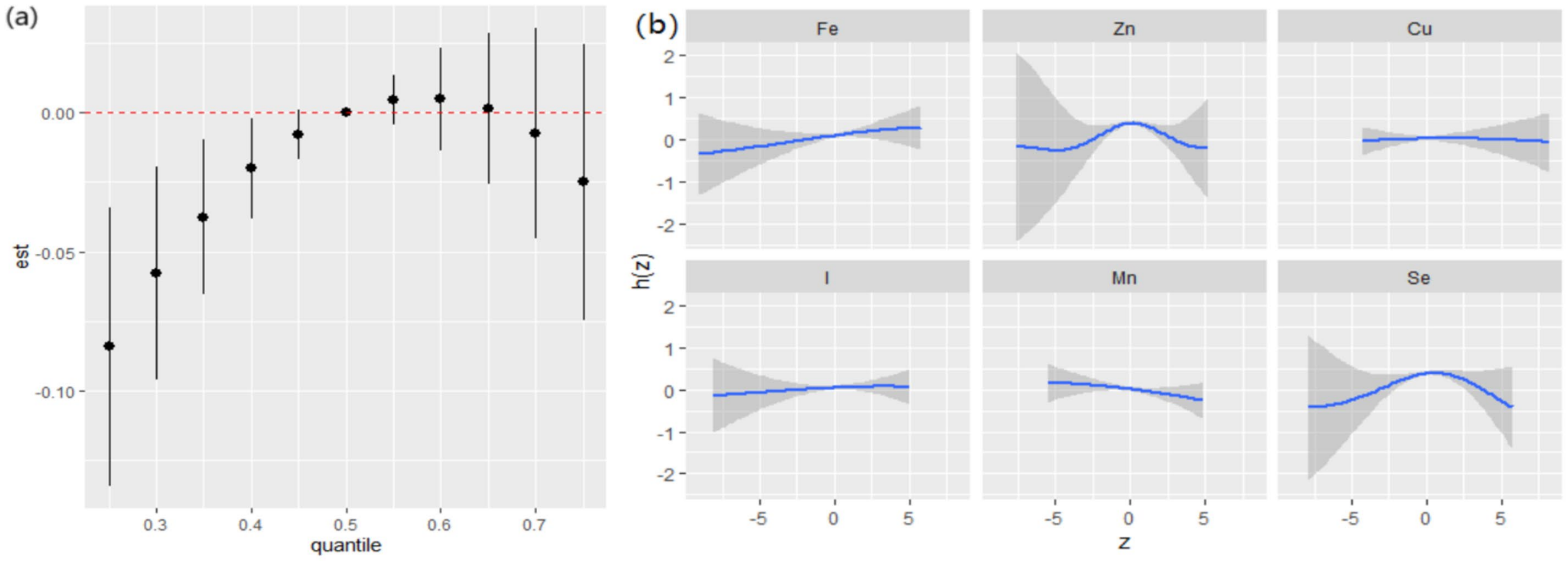
Joint associations between ETEs and general cognition, estimated using Bayesian Kernel Machine Regression (BKMR) in the hypertension group. **(a)** Overall effects of dietary ETEs on general cognition. **(b)** Univariate exposure response function and 95% credible interval (shaded areas) for each dietary ETE, when other ETEs were fixed at their 50th percentile. Adjusted for age, sex, ethnicity, smoking, alcohol, BMI, TDI, physical activity, total energy, diabetes, hypertension, cardiovascular disease, education, and employment.

Although significant interactions between dietary ETEs and hypertension were not observed (Supplementary Table S5), given that hypertension is a prominent risk factor for cognitive decline, we also conducted a subgroup analysis by hypertension. In the hypertensive group, dietary ETEs were significantly associated with a decline in general cognition when ETEs were below the 40th percentile (Figure 7a). The highest PIP was Zn (1.000), followed by Se (0.821), indicating that Zn and Se played relatively more significant roles in the effects of the ETE mixture on general cognition. In the univariate exposure–response analysis, non-linear associations were observed between Zn, Se, and general cognition (Figure 7b). In the single exposure-effect result, an increase in dietary Mn intake was associated with a decline in general cognition when the other dietary ETEs were fixed at the 25th percentile (Supplementary Figure S6).

The results of the sensitivity analysis were similar to those of the main analysis (Supplementary Figures S7–S11), indicating the robustness of the study findings.

## Discussion

Based on the dietary and cognitive test data from the UKB, the results of RCS analyses indicated that Fe, Zn, Cu, I, Mn, and Se showed inverted “U”-shaped association with general cognition, with inflection points at approximately 15 mg/day of Fe, 10 mg/day of Zn, 1.5 mg/day of Cu, 250 μg/day of I, 5 mg/day of Mn, and 45 μg/day of Se. The inflection points of these ETEs were similar to the Recommended Dietary Allowances (RDAs).^1^ Therefore, the dietary intake of ETEs must reach the corresponding RDAs.

The BKMR results, consistent with RCS, reported that the dietary ETE mixture showed an inverted “U”-shaped association with general cognition, with Fe and Zn playing major roles. With the increase in single dietary ETE and its mixture intake, general cognition showed a trend of first increasing and then decreasing. We also found interactions among dietary Fe, Mn, and Zn. Dietary Mn was the major contributor to the impact of dietary ETEs on general cognition in males, while in females, it was Zn. In the hypertensive population, dietary Zn and Mn played major roles.

Our study indicated that the dietary mixture of ETEs and single dietary ETEs showed an inverted “U”-shaped association with general cognition. Previous studies have indicated that inadequate dietary Fe intake is linked to cognitive decline, while excessive dietary Fe intake is also significantly associated with cognitive decline (14, 36). Similarly, both excessive and insufficient dietary intake of Zn, Cu, I, Mn, and Se are negatively associated with cognition (8, 17, 37–41). Previous BKMR studies on the impact of whole blood and urine ETE mixture on cognition in Chinese populations have found a linear positive correlation, which is inconsistent with the results of our study (28, 29). Apart from differences in the study population and outcome definition, this discrepancy could be due to variations in the sources and composition of ETEs and interaction among ETEs during absorption. In the previous studies, ETEs were sourced from urine and whole blood, whereas in this study, ETEs were sourced from the diet. Besides, the ETEs in previous studies were chromium (Cr), Se, Mn, and Cu in whole blood, and Se, vanadium (V), cobalt (Co), strontium (Sr), and molybdenum (Mo) in urine, while the dietary ETEs in our study were Fe, Zn, Cu, I, Mn, and Se.

When the intake of dietary ETEs was below the 50th percentile, general cognition increased with increasing intake. The possible explanation for this is that Fe is essential for the synthesis of hemoglobin, which is crucial for oxygen transport in the blood. Adequate Fe intake is beneficial for preventing anemia, ensuring an adequate oxygen supply to the brain, and thereby protecting cognition (42). Ensuring a moderate Zn intake is necessary for maintaining a normal immune system and antioxidant system functions (37, 43). In addition to maintaining redox homeostasis and inflammation, Cu is also involved in maintaining brain functions such as neurotransmitter synthesis (44). Iodine (I) is an essential component of thyroid hormones, so ensuring an adequate intake of I is crucial for maintaining thyroid health, which is closely related to cognition (41). Mn, as a coenzyme, participates in maintaining cognitive processes, including energy metabolism, antioxidant systems, brain ammonia clearance, and neurotransmitter synthesis (17, 45). Se exerts cognitive protection through the synthesis of selenoprotein. Glutathione peroxidase is an important selenoprotein that is believed to protect neurons from oxidative stress-induced damage due to its antioxidant activity (11, 46).

Although moderate levels of Fe, Zn, Cu, I, Mn, and Se play important roles in maintaining cognitive health, they also exhibit toxicity when consumed in excess. Excessive Fe intake may result in iron deposition in the brain, which can affect cognitive function by influencing protein misfolding and increasing oxidative stress in the brain (47, 48). An imbalance in Zn homeostasis may lead to chronic inflammation and oxidative stress, resulting in cognitive decline (49, 50). Excessive Cu can bind to amyloid-*β* to produce reactive oxygen species, leading to oxidative stress (51), and the generation of ceruloplasmin, which increases the risk of Alzheimer’s disease (52). Excessive intake of I can precipitate thyroid disorders such as hyperthyroidism. Thyroid disorders can impact cognitive function by affecting the conversion of thyroxine (T4) to triiodothyronine (T3) in the central nervous system (CNS) (53, 54). Superfluous Mn can accumulate in the cerebral cortex and hippocampus (55, 56), and the neurotoxic effects of Mn, such as oxidative stress, glutamate excitotoxicity, protein misfolding, and inflammation, can contribute to cognitive impairment (57). Besides, excessive Se can induce oxidative stress and alter the regulation of genes involved in dopamine neurotransmission, leading to cognitive impairment (58, 59).

We observed differences in RCS results and the factors that played a major role in the association between dietary ETE mixture and general cognition. Mn played a major role in males, while Zn, in females. The biological mechanisms underlying these differences are currently unclear but could be related to the modulatory effects of sex hormones (60). Further studies are needed to elucidate the possible mechanisms.

This study has several strengths. First, a large-sample prospective cohort data was used to investigate the inverted U-shaped associations of ETEs and their mixture with cognition, finding that an appropriate amount is beneficial for cognition. Second, major elements in the associations between ETE mixture and cognition were explored, revealing sex differences in major elements and providing a basis for differentiated supplementation of trace elements. Third, the inflection points for the association between ETEs and cognitive function we found were approximately close to the RDAs, which provides new evidence for determining RDAs. Furthermore, we conducted multiple sensitivity analyses and obtained similar results, demonstrating the robustness of this study.

However, this study also has some limitations. First, the data of dietary ETEs were self-reported through the 24-h dietary recall questionnaire, which could be subject to recall bias and information distortion. Second, although we have adjusted many confounding factors, there could still be unmeasured confounding factors that could affect the study results. Third, this study had a relatively short follow-up period. Future studies could further explore the association between the ETEs’ dynamic changes and cognitive changes over a longer follow-up period. Fourth, this study implemented strict criteria during the screening of participants. The exclusion of individuals with missing data on cognition and/or covariates could introduce selection bias and affect the generalizability of findings. Finally, although we have used more cognitive test results to redefine the outcome of general cognition to enhance robustness, our results could lack comparability with previous studies.

## Conclusion

In summary, Fe, Zn, Cu, I, Mn, and Se, as well as their mixture, show an inverted “U”-shaped relationship with general cognition. Fe and Zn play important roles in the mixture within the general population. In males, Mn is the primary factor, while in females and hypertensive patients, the effect of Zn is more significant. Our study emphasizes the importance of moderate intake of ETEs in maintaining cognitive health, while also warning of the potential harm of excessive intake on cognition. Therefore, it is recommended that people control their intake of ETEs in their daily diet to delay cognitive decline.

## Data Availability

Publicly available datasets were analyzed in this study. This data can be found here: the UK Biobank (www.ukbiobank.ac.uk/).

## Footnotes

^1^ https://www.efsa.europa.eu/en/topics/topic/dietary-reference-values
