# Supplementary material for: The association of essential dietary trace elements and their mixture with cognition: a prospective study
Source: Front Nutr. 2025 Jul 4;12:1461852. doi: 10.3389/fnut.2025.1461852 (PMC12270852; doi:10.3389/fnut.2025.1461852)
Supplement: Supplementary file 1 [file Data_Sheet_1.DOCX]

Table S1. Cognitive domain, definition, and Field ID details for cognitive function tests

| **Cognitive function tests** | **Cognitive domain** | **definition** | **transformed** | **Field ID** |
| --- | --- | --- | --- | --- |
| Prospective memory test | prospective memory | the initial chose was correct | / | 20018 |
| Pairs matching test | visual declarative memory | number of incorrect matches in round | log(x+1) | 399 |
| Numeric memory test | working memory | maximum digits remembered correctly | / | 4282 |
| Fluid Intelligence test | verbal and numerical reasoning | number of fluid intelligence questions attempted within time limit | / | 20128 |
| Reaction time test | processing speed | mean time to correctly identify matches | log | 20023 |
| Trail making test 2 | executive function | duration to complete alphanumeric path | log | 6350 |
| Symbol digit substitution test | processing speed | number of symbol digit matches made correctly | / | 23324 |
| Picture vocabulary test | vocabulary | vocabulary level | / | 6364 |
| Paired associate learning test | visual declarative memory | number of word pairs correctly associated | / | 20197 |
| Matrix pattern completion test | non-verbal reasoning | number of puzzles correctly solved | / | 6373 |
| Tower rearranging test | executive function | number of puzzles correct | / | 21004 |

Table S2. Definitions of hypertension, stroke, coronary heart disease, and diabetes

| **Diseases** | **Self-reported Information** | **ICD-9 information** | **ICD-10 information** | **OPCS-4 information** |
| --- | --- | --- | --- | --- |
| Hypertension | 2966, 6150 (4), 6153 (2), 6177 (2), 20002 (1065, 1072), 20008, 20009, 20010, 20011 | 41271 (401-405) 41281 | 41270 (I10-I13, I15, O10), 41280, 131292, 131290, 131288, 131286, 131294, 132180, 131295, 131293, 131291, 131289, 131287, 132181 |  |
| Stroke | 6150 (3), 4056, 20002 (1081, 1491, 1583, 1086), 20008, 20009, 20010, 20011 | 41271 (3361, 3623, 430, 431, 4329, 4330, 4331, 4332,4333, 4338, 4339, 434, 436), 41281 | 41270 (I60, I61, I629, I63, I64, I678, I690, I693, G951, H341, H342, S066), 41280, 131378, 131376, 131374, 131372, 131370, 131368, 131366, 131364, 131362, 131360, 131180, 131379, 131377, 131375, 131373, 131371, 131369, 131367, 131365, 131363, 131361, 131181 | 41272 (A052- A054, L351, L353, L343), 41282 |
| Diabetes | 2443 (1), 2976, 6153 (3), 6177(3), 20002 (1220, 1222, 1223), 20008, 20009 | 41271 (250, 3572, 3620), 41281 | 41270 (E10-E14, G590, G632, H280, H360, M142, N083) 41280, 130714, 130712, 130710, 130708, 130706, 130715, 130713, 130711, 130709, 130707 |  |

ICD: International Classification of Diseases

the Office of Population Censuses and Surveys Classification of Interventions and Procedures, version 4

Table S3. Associations between single dietary essential trace element intake and cognitive domains

| Dietary essential trace elements | Cognitive domains | | | | | | | | |
| --- | --- | --- | --- | --- | --- | --- | --- | --- | --- |
|  | Pairs matching |  | Numeric memory |  | Prospective memory |  | Reaction time |  | Fluid intelligence / reasoning |
|  | *β (95%CI)* |  | *β (95%CI)* |  | *β (95%CI)* |  |  |  |  |
| Fe |  |  |  |  |  |  |  |  |  |
| Quartiles 1 | reference |  | reference |  | reference |  | reference |  | reference |
| Quartiles 2 | -0.008(-0.032, 0.016) |  | **0.070(0.022, 0.118)** |  | **-0.188(-0.312, -0.063)** |  | -0.002(-0.004, 0.001) |  | **-0.011(-0.018, -0.005)** |
| Quartiles 3 | -0.005(-0.031, 0.021) |  | **0.075(0.023, 0.126)** |  | -0.045(-0.175, 0.085) |  | **0.008(0.007, 0.008)** |  | -0.005(-0.012, 0.001) |
| Quartiles 4 | 0.003(-0.028, 0.034) |  | **0.064(0.001, 0.126)** |  | -0.026(-0.183, 0.131) |  | **-0.042(-0.047, -0.037)** |  | -0.008(-0.016, 0.001) |
| Zn |  |  |  |  |  |  |  |  |  |
| Quartiles 1 | reference |  | reference |  | reference |  | reference |  | reference |
| Quartiles 2 | -0.015(-0.039, 0.008) |  | **0.066(0.018, 0.113)** |  | -0.104(-0.225, 0.017) |  | -0.002(-0.009, 0.003) |  | **0.084(0.007, 0.161)** |
| Quartiles 3 | -0.013(-0.038, 0.013) |  | **0.016(0.023, 0.126)** |  | **-0.214(-0.344, -0.085)** |  | -0.003(-0.010, 0.008) |  | 0.161(-0.012, 0.033) |
| Quartiles 4 | 0.003(-0.028, 0.034) |  | -0.020(-0.078, 0.038) |  | -0.054(-0.183, 0.093) |  | 0.005(-0.047, 0.013) |  | -0.042(-0.135, 0.052) |
| Cu |  |  |  |  |  |  |  |  |  |
| Quartiles 1 | reference |  | reference |  | reference |  | reference |  | reference |
| Quartiles 2 | -0.007(-0.031, 0.017) |  | 0.035(-0.014, 0.083) |  | -0.120(-0.242, 0.001) |  | -0.002(-0.008, 0.003) |  | 0.075(-0.003, 0.152) |
| Quartiles 3 | 0.002(-0.023, 0.028) |  | **0.081(0.023, 0.126)** |  | **-0.240(-0.372, -0.108)** |  | -0.006(-0.010, 0.008) |  | 0.152(-0.012, 0.058) |
| Quartiles 4 | 0.027(-0.028, 0.034) |  | 0.013(-0.047, 0.072) |  | **-0.178(-0.329, -0.027)** |  | -0.003(-0.047, 0.005) |  | 0.015(-0.016, 0.058) |
| I |  |  |  |  |  |  |  |  |  |
| Quartiles 1 | reference |  | reference |  | reference |  | reference |  | reference |
| Quartiles 2 | 0.009(-0.015, 0.032) |  | 0.042(-0.005, 0.089) |  | -0.086(-0.208, 0.036) |  | 0.001(-0.006, 0.003) |  | 0.065(-0.011, 0.141) |
| Quartiles 3 | 0.005(-0.023, 0.028) |  | **0.012(0.023, 0.126)** |  | -0.038(-0.162, 0.086) |  | 0.001(-0.010, 0.008) |  | 0.141(-0.012, 0.093) |
| Quartiles 4 | 0.029(-0.028, 0.034) |  | **0.051(0.000, 0.102)** |  | 0.014(-0.115, 0.142) |  | -0.001(-0.047, 0.006) |  | 0.059(-0.016, 0.093) |
| Mn |  |  |  |  |  |  |  |  |  |
| Quartiles 1 | reference |  | reference |  | reference |  | reference |  | reference |
| Quartiles 2 | -0.010(-0.034, 0.013) |  | **0.000(-0.047, 0.047)** |  | -0.099(-0.220, 0.022) |  | 0.001(-0.006, 0.003) |  | 0.017(-0.059, 0.093) |
| Quartiles 3 | 0.008(-0.023, 0.028) |  | **0.024(0.023, 0.126)** |  | -0.088(-0.213, 0.037) |  | 0.001(-0.010, 0.008) |  | 0.093(-0.012, 0.656) |
| Quartiles 4 | 0.023(-0.028, 0.034) |  | -0.011(-0.065, 0.043) |  | -0.031(-0.167, 0.105) |  | 0.002(-0.047, 0.009) |  | -0.041(-0.127, 0.052) |
| Se |  |  |  |  |  |  |  |  |  |
| Quartiles 1 | reference |  | reference |  | reference |  | reference |  | reference |
| Quartiles 2 | -0.020(-0.044, 0.003) |  | **0.101(0.054, 0.148)** |  | **-0.144(-0.265, -0.024)** |  | -0.003(-0.009, 0.003) |  | **0.168(0.093, 0.244)** |
| Quartiles 3 | -0.014(-0.023, 0.028) |  | **0.058(1.023, 1.126)** |  | **-0.158(-0.281, -0.035)** |  | -0.003(-0.010, 0.008) |  | 0.244(-1.012, 0.000) |
| Quartiles 4 | -0.009(-0.028, 0.034) |  | **0.054(0.004, 0.104)** |  | 0.013(-0.111, 0.137) |  | -0.001(-0.047, 0.005) |  | 0.051(-0.029, 0.130) |

Table S4. Posterior inclusion probabilities (PIPs) of sex dietary essential trace elements

| Dietary essential trace elements | PIPs | | | | | | | | | | | | | | |
| --- | --- | --- | --- | --- | --- | --- | --- | --- | --- | --- | --- | --- | --- | --- | --- |
|  | Total |  | Male |  | Female |  | Hypertension |  | Healthy group |  | White |  | General cognition (11)^a^ |  | Not-dementia ^b^ |
| Fe | 1.000 |  | 0.985 |  | 0.987 |  | 0.208 |  | 0.999 |  | 1.000 |  | 0.737 |  | 1.000 |
| Zn | 1.000 |  | 0.971 |  | 1.000 |  | 1.000 |  | 1.000 |  | 1.000 |  | 1.000 |  | 1.000 |
| Cu | 0.980 |  | 0.983 |  | 0.135 |  | 0.164 |  | 0.994 |  | 0.984 |  | 0.893 |  | 0.980 |
| I | 0.056 |  | 0.559 |  | 0.054 |  | 0.207 |  | 0.284 |  | 0.681 |  | 0.117 |  | 0.091 |
| Mn | 0.998 |  | 1.000 |  | 0.730 |  | 0.218 |  | 1.000 |  | 1.000 |  | 0.597 |  | 1.000 |
| Se | 0.954 |  | 0.952 |  | 0.476 |  | 0.821 |  | 0.820 |  | 0.987 |  | 1.000 |  | 0.972 |

Note. a, General cognition (11), General cognition based on the 11 cognitive tests from the UK Biobank

b, Not-dementia, exclude participants who developed dementia in the first ten years of follow-up

c, dementia as the outcome variable

Table S5. Results of the interaction effects of sex dietary essential trace elements with sex, and hypertension

| ETEs | interaction effects | *β* | *P* |
| --- | --- | --- | --- |
| Fe | sex | -8.533E-03 | 0.604 |
|  | hypertension | -1.343E-02 | 0.484 |
| Zn | sex | 9.245E-03 | 0.573 |
|  | hypertension | 4.208E-03 | 0.826 |
| Cu | sex | -1.799E-02 | 0.269 |
|  | hypertension | -1.077E-02 | 0.575 |
| Mn | sex | -4.349E-02 | 0.007 |
|  | hypertension | -2.643E-03 | 0.891 |
| Se | sex | 3.376E-03 | 0.834 |
|  | hypertension | 3.147E-03 | 0.869 |
| I | sex | 1.499E-02 | 0.356 |
|  | hypertension | 7.986E-03 | 0.680 |


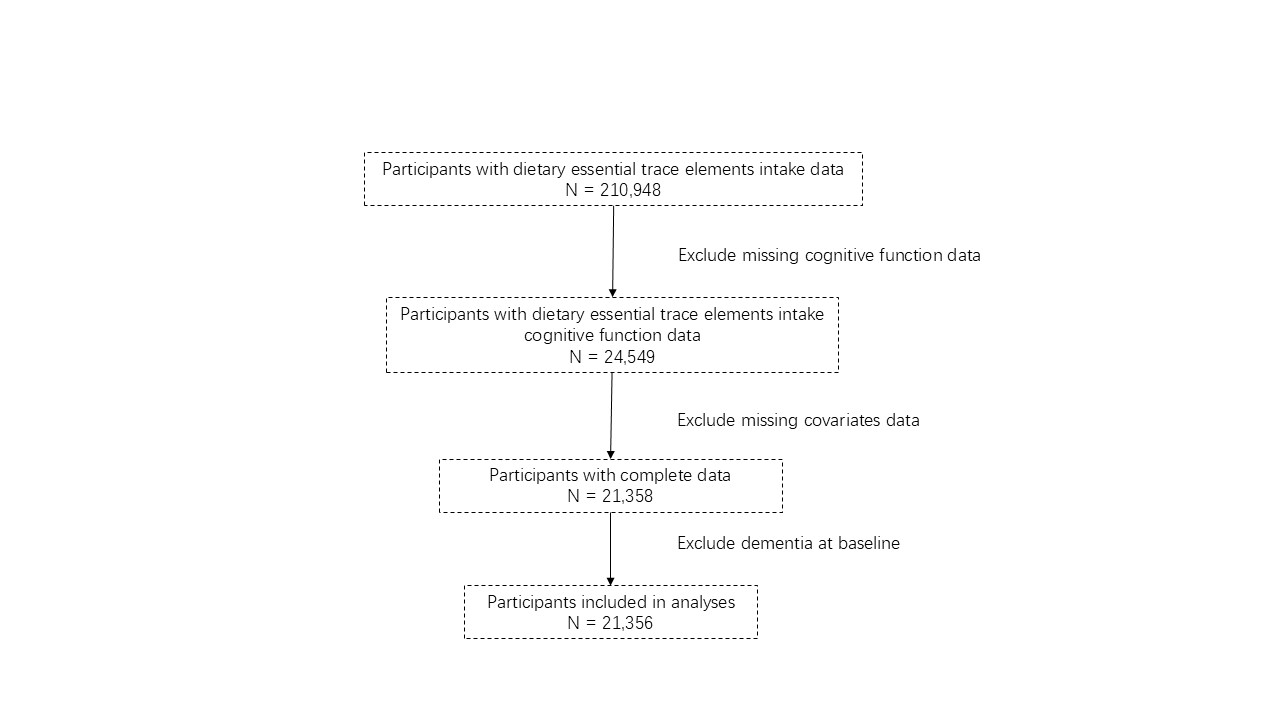


Figure S1. Flowchart illustrating criteria for selection of samples in the current study.


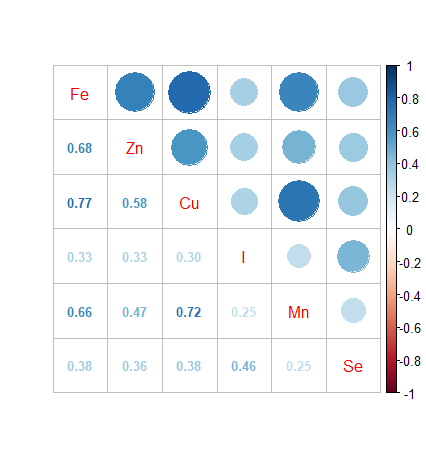


Figure S2. Pairwise Spearman correlation coefficients among dietary ETEs intakes measured in the participants. All the correlations were statistically significant (P < 0.0001). Dietary ETEs intakes were log(x+1)-transformed and standardization; Fe, iron; Zn, zinc; Cu, copper; I, iodine; Mn, Manganese; Se, selenium.


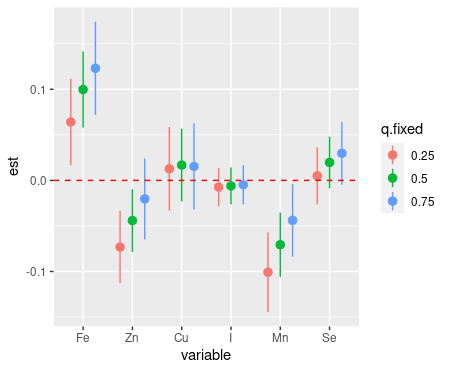


Figure S3. Single-exposure effect of each dietary ETEs intake on general cognition. The red, green, or blue dots with the corresponding error bars represented the estimated changes and 95% credible intervals associated with an interquartile range change in each dietary ETEs intake. Dietary ETEs intakes were log(x+1)-transformed and standardization.


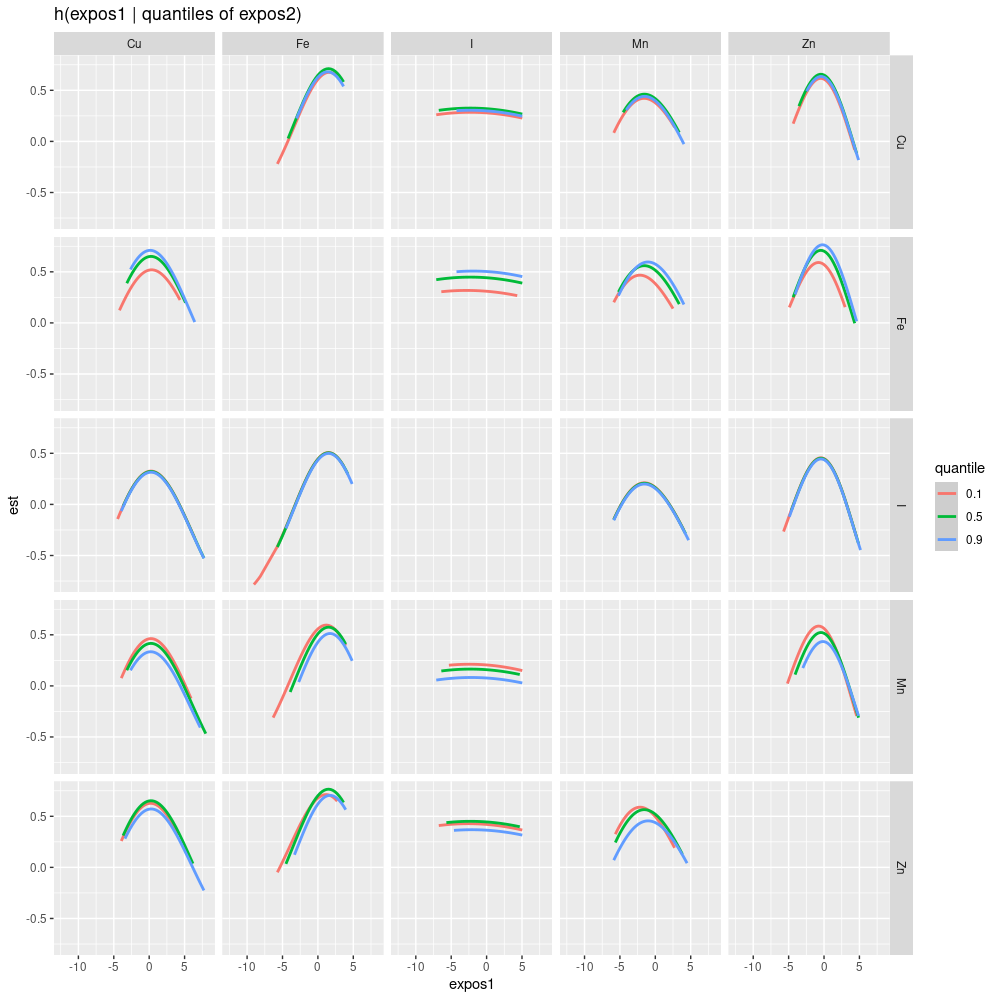


Figure S4. Bivariate exposure-response functions for each ETE when the second ETE was fixed at the different level (10th, 50th, and 90th percentiles) and the remaining ETEs were fixed at their medians. Dietary ETEs intakes were log(x+1)-transformed and standardization.


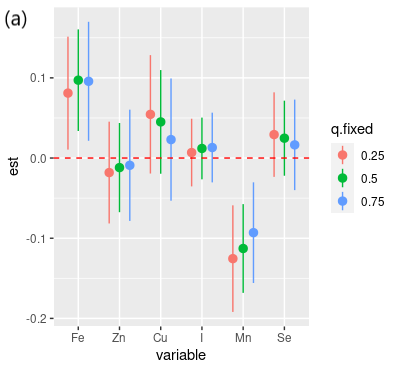

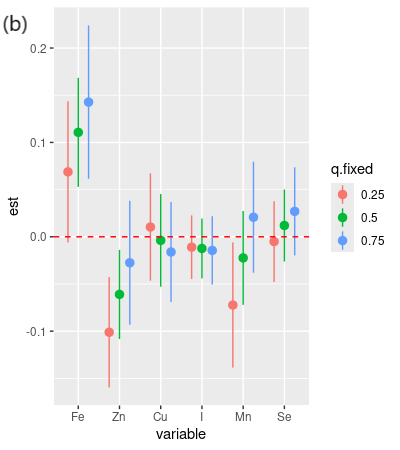


Figure S5. Single-exposure effect of each dietary ETEs intake on general cognition stratified by sex (a, males; b, females). The red, green, or blue dots with the corresponding error bars represented the estimated changes and 95% credible intervals associated with an interquartile range change in each dietary ETEs intake. Dietary ETEs intakes were log(x+1)-transformed and standardization.


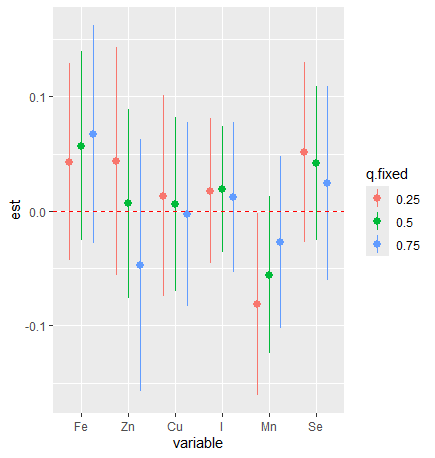


Figure S6. Single-exposure effect of each dietary ETEs intake on general cognition in hypertension group. The red, green, or blue dots with the corresponding error bars represented the estimated changes and 95% credible intervals associated with an interquartile range change in each dietary ETEs intake. Dietary ETEs intakes were log(x+1)-transformed and standardization.


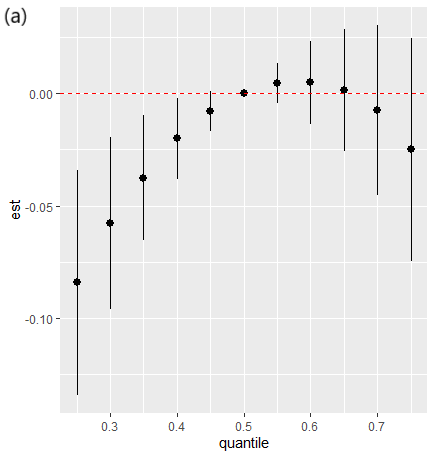

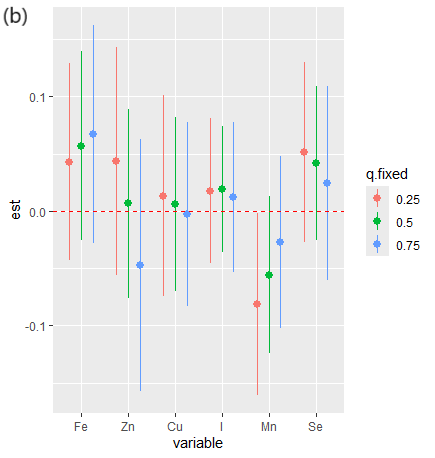


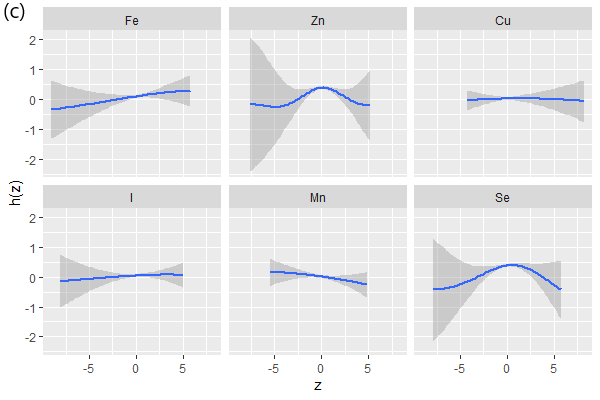


Figure S7. Joint associations between ETEs and general cognition, estimated using Bayesian kernel machine regression in hypertension group. (a) Overall effects of dietary ETEs on general cognition. These values indicated the change in general cognition when all dietary ETEs were at different percentiles compared to when they were fixed at their 50th percentile. (b) Single-exposure effect of each dietary ETEs intake on general cognition. The red, green, or blue dots with the corresponding error bars represented the estimated changes and 95% credible intervals associated with an interquartile range change in each dietary ETEs intake. (c) Univariate exposure-response function and 95% credible interval (shaded areas) for each dietary ETEs, when other ETEs were fixed at their 50th percentile. Dietary ETEs intakes were log(x+1)-transformed and standardization.


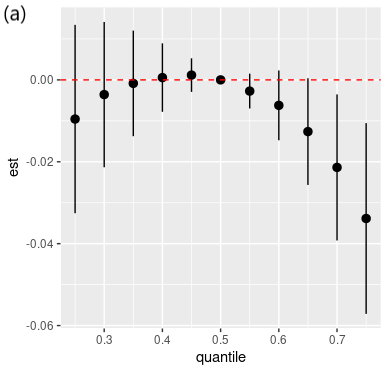

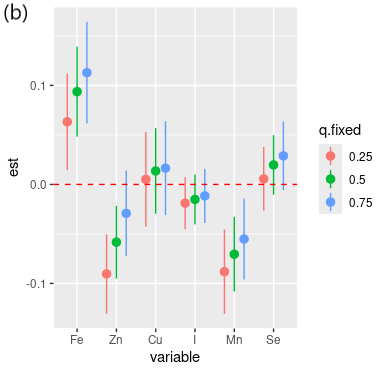


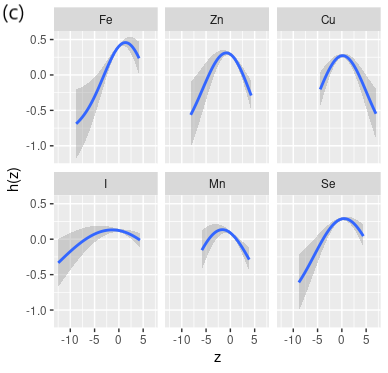


Figure S8 Joint associations between ETEs and general cognition, estimated using Bayesian kernel machine regression after excluded individuals with hypertension, diabetes, and cardiovascular diseases. (a) Overall effects of dietary ETEs on general cognition. These values indicated the change in general cognition when all dietary ETEs were at different percentiles compared to when they were fixed at their 50th percentile. (b) Single-exposure effect of each dietary ETEs intake on general cognition. The red, green, or blue dots with the corresponding error bars represented the estimated changes and 95% credible intervals associated with an interquartile range change in each dietary ETEs intake. (c) Univariate exposure-response function and 95% credible interval (shaded areas) for each dietary ETEs, when other ETEs were fixed at their 50th percentile. Dietary ETEs intakes were log(x+1)-transformed and standardization.


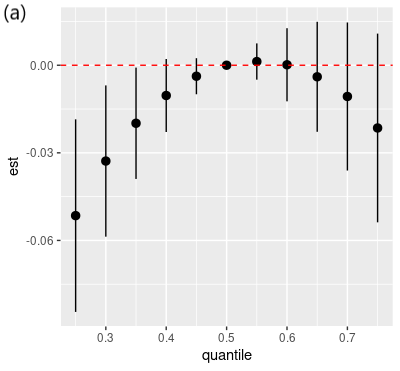

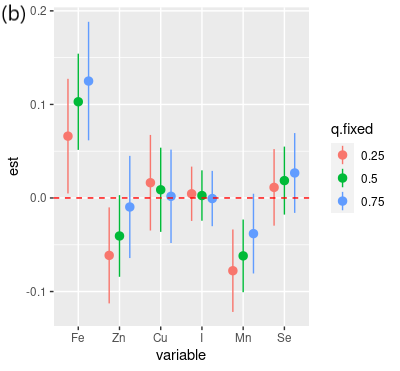


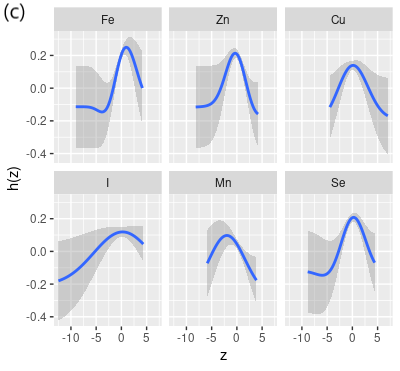


Figure S9 Joint associations between ETEs and general cognition, estimated using Bayesian kernel machine regression after excluded not-white individuals. (a) Overall effects of dietary ETEs on general cognition. These values indicated the change in general cognition when all dietary ETEs were at different percentiles compared to when they were fixed at their 50th percentile. (b) Single-exposure effect of each dietary ETEs intake on general cognition. The red, green, or blue dots with the corresponding error bars represented the estimated changes and 95% credible intervals associated with an interquartile range change in each dietary ETEs intake. (c) Univariate exposure-response function and 95% credible interval (shaded areas) for each dietary ETEs, when other ETEs were fixed at their 50th percentile. Dietary ETEs intakes were log(x+1)-transformed and standardization.


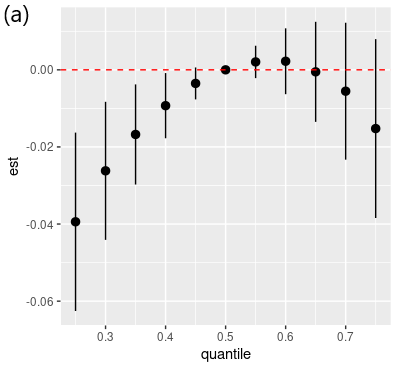

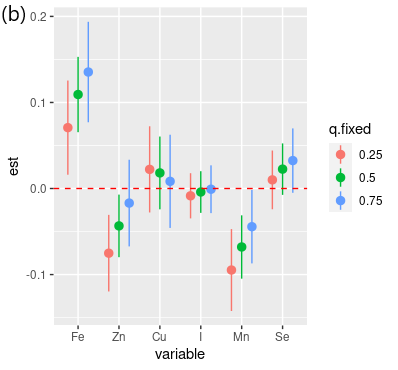


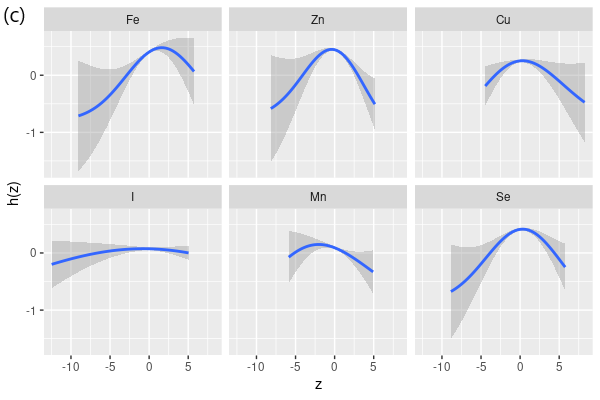


Figure S10. Joint associations between ETEs and general cognition, estimated using Bayesian kernel machine regression after excluded participants who development dementia in the follow-up period. (a) Overall effects of dietary ETEs on general cognition. These values indicated the change in general cognition when all dietary ETEs were at different percentiles compared to when they were fixed at their 50th percentile. (b) Single-exposure effect of each dietary ETEs intake on general cognition. The red, green, or blue dots with the corresponding error bars represented the estimated changes and 95% credible intervals associated with an interquartile range change in each dietary ETEs intake. (c) Univariate exposure-response function and 95% credible interval (shaded areas) for each dietary ETEs, when other ETEs were fixed at their 50th percentile. Dietary ETEs intakes were log(x+1)-transformed and standardization.


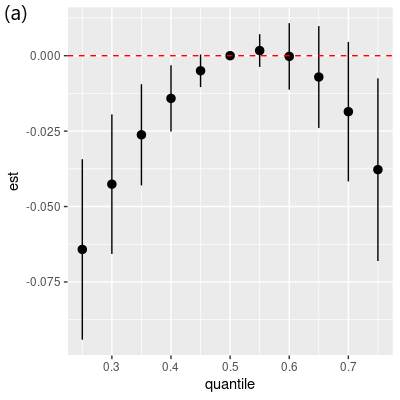

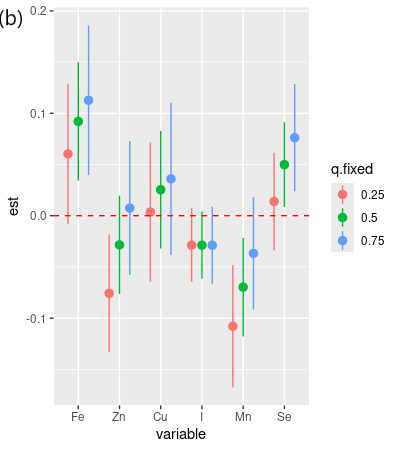


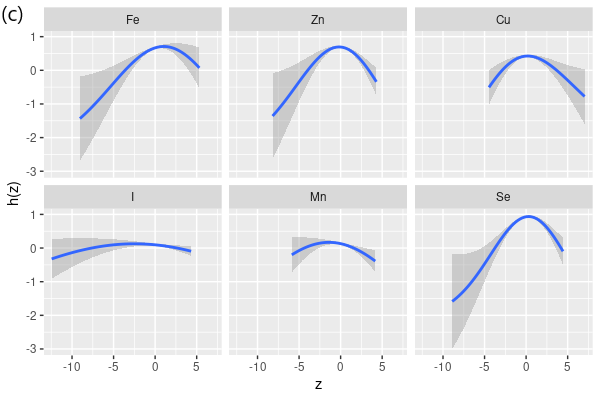


Figure S11. Joint associations between ETEs and general cognition which defined by 11 cognitive tests, estimated using Bayesian kernel machine regression. (a) Overall effects of dietary ETEs on general cognition. These values indicated the change in general cognition when all dietary ETEs were at different percentiles compared to when they were fixed at their 50th percentile. (b) Single-exposure effect of each dietary ETEs intake on general cognition. The red, green, or blue dots with the corresponding error bars represented the estimated changes and 95% credible intervals associated with an interquartile range change in each dietary ETEs intake. (c) Univariate exposure-response function and 95% credible interval (shaded areas) for each dietary ETEs, when other ETEs were fixed at their 50th percentile. Dietary ETEs intakes were log(x+1)-transformed and standardization.
